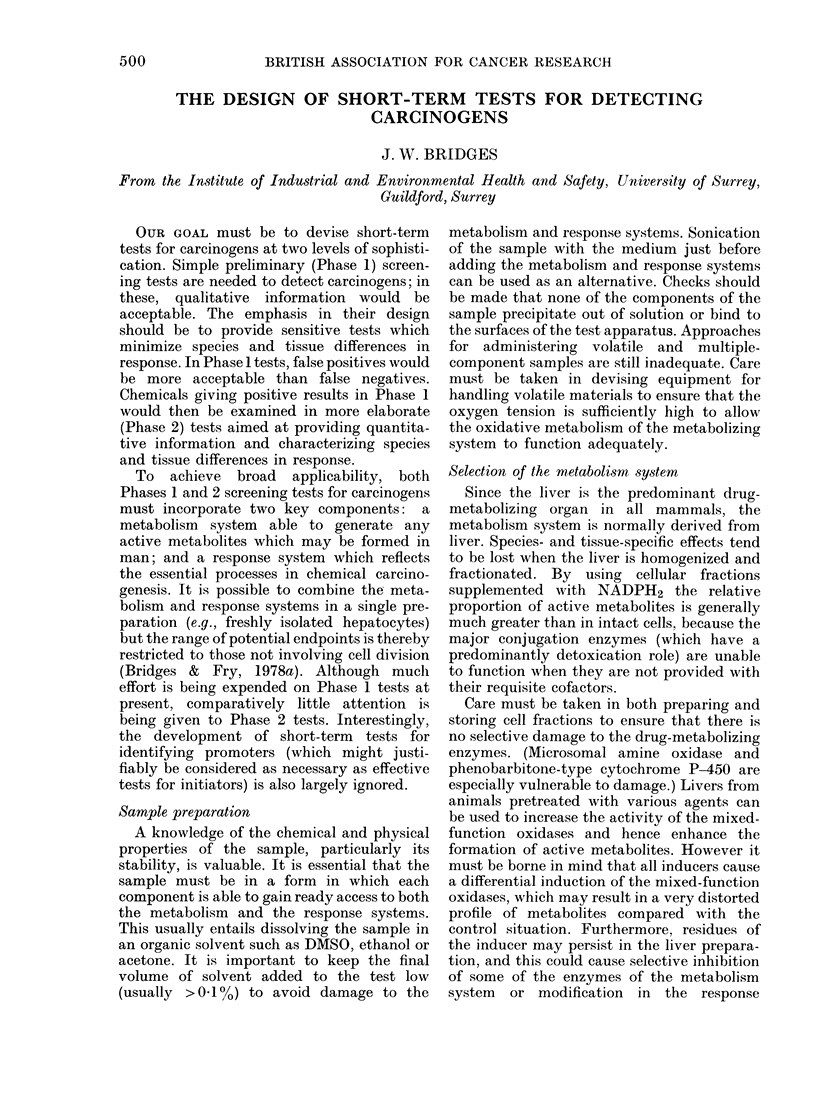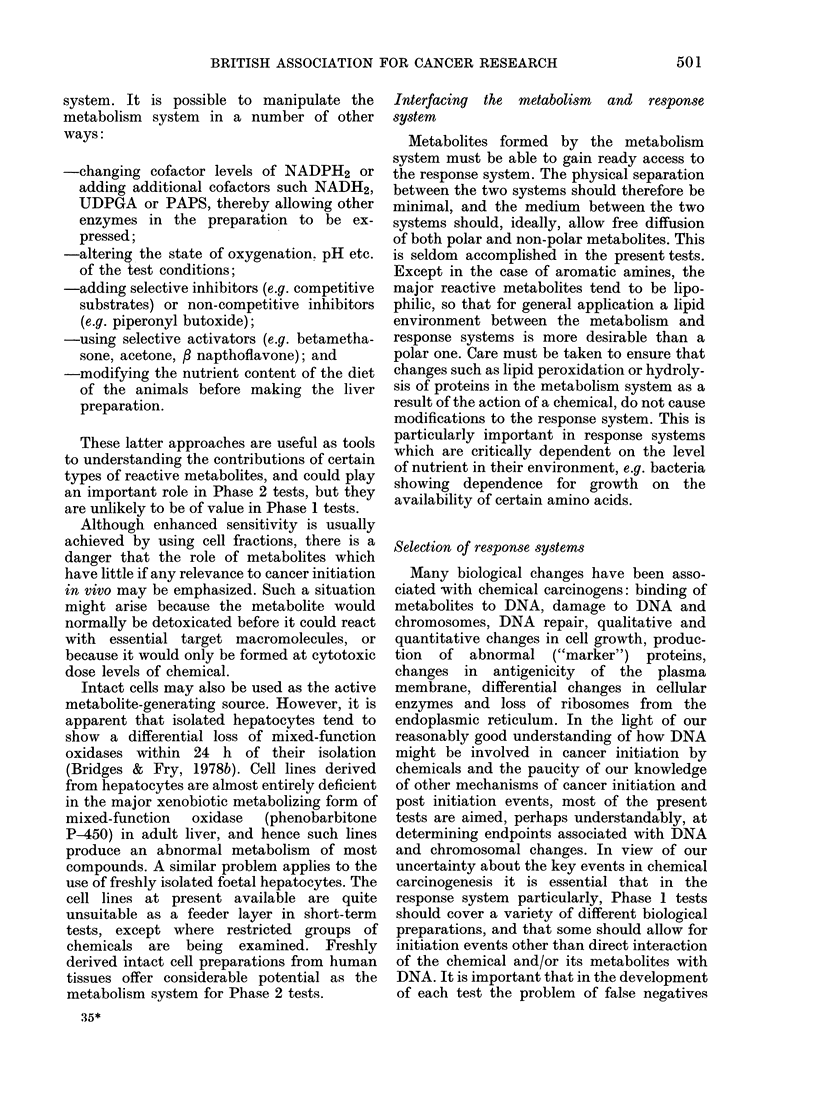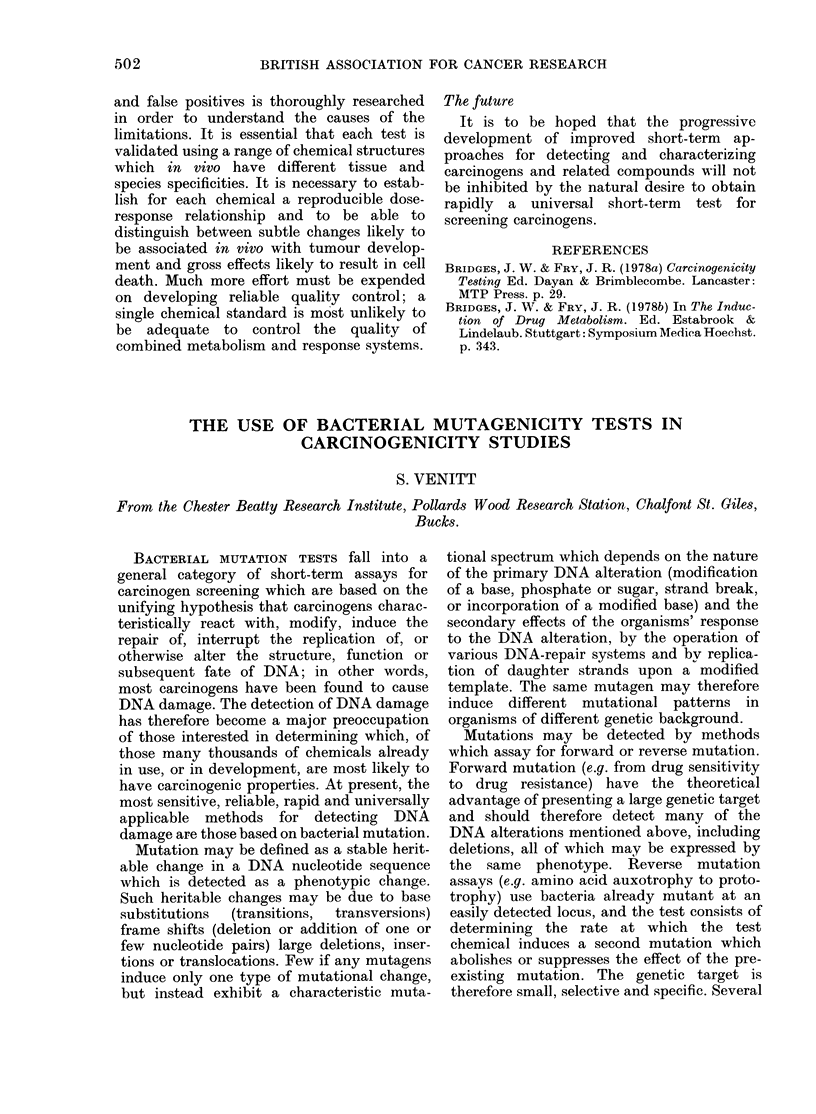# The design of short-term tests for detecting carcinogens.

**DOI:** 10.1038/bjc.1980.81

**Published:** 1980-03

**Authors:** J. W. Bridges


### THE DESIGN OF SHORT-TERM TESTS FOR DETECTING CARCINOGENS

J. W. BRIDGES

*From the Institute of Industrial and Environmental Health and Safety, University of Surrey, Guildford, Surrey*

OUR GOAL must be to devise short-term tests for carcinogens at two levels of sophistication. Simple preliminary (Phase 1) screening tests are needed to detect carcinogens; in these, qualitative information would be acceptable. The emphasis in their design should be to provide sensitive tests which minimize species and tissue differences in response. In Phase 1 tests, false positives would be more acceptable than false negatives. Chemicals giving positive results in Phase 1 would then be examined in more elaborate (Phase 2) tests aimed at providing quantitative information and characterizing species and tissue differences in response.

To achieve broad applicability, both Phases 1 and 2 screening tests for carcinogens must incorporate two key components: a metabolism system able to generate any active metabolites which may be formed in man; and a response system which reflects the essential processes in chemical carcinogenesis. It is possible to combine the metabolism and response systems in a single preparation (*e.g.*, freshly isolated hepatocytes) but the range of potential endpoints is thereby restricted to those not involving cell division (Bridges & Fry, 1978*a*). Although much effort is being expended on Phase 1 tests at present, comparatively little attention is being given to Phase 2 tests. Interestingly, the development of short-term tests for identifying promoters (which might justifiably be considered as necessary as effective tests for initiators) is also largely ignored.

*Sample preparation*

A knowledge of the chemical and physical properties of the sample, particularly its stability, is valuable. It is essential that the sample must be in a form in which each component is able to gain ready access to both the metabolism and the response systems. This usually entails dissolving the sample in an organic solvent such as DMSO, ethanol or acetone. It is important to keep the final volume of solvent added to the test low (usually $>0{\cdot}1\%$) to avoid damage to the metabolism and response systems. Sonication of the sample with the medium just before adding the metabolism and response systems can be used as an alternative. Checks should be made that none of the components of the sample precipitate out of solution or bind to the surfaces of the test apparatus. Approaches for administering volatile and multiple-component samples are still inadequate. Care must be taken in devising equipment for handling volatile materials to ensure that the oxygen tension is sufficiently high to allow the oxidative metabolism of the metabolizing system to function adequately.

*Selection of the metabolism system*

Since the liver is the predominant drug-metabolizing organ in all mammals, the metabolism system is normally derived from liver. Species- and tissue-specific effects tend to be lost when the liver is homogenized and fractionated. By using cellular fractions supplemented with $NADPH_2$ the relative proportion of active metabolites is generally much greater than in intact cells, because the major conjugation enzymes (which have a predominantly detoxication role) are unable to function when they are not provided with their requisite cofactors.

Care must be taken in both preparing and storing cell fractions to ensure that there is no selective damage to the drug-metabolizing enzymes. (Microsomal amine oxidase and phenobarbitone-type cytochrome P–450 are especially vulnerable to damage.) Livers from animals pretreated with various agents can be used to increase the activity of the mixed-function oxidases and hence enhance the formation of active metabolites. However it must be borne in mind that all inducers cause a differential induction of the mixed-function oxidases, which may result in a very distorted profile of metabolites compared with the control situation. Furthermore, residues of the inducer may persist in the liver preparation, and this could cause selective inhibition of some of the enzymes of the metabolism system or modification in the response system. It is possible to manipulate the metabolism system in a number of other ways:

—changing cofactor levels of $NADPH_2$ or adding additional cofactors such $NADH_2$, UDPGA or PAPS, thereby allowing other enzymes in the preparation to be expressed;
—altering the state of oxygenation, pH etc. of the test conditions;
—adding selective inhibitors (*e.g.* competitive substrates) or non-competitive inhibitors (*e.g.* piperonyl butoxide);
—using selective activators (*e.g.* betamethasone, acetone, $\beta$ napthoflavone); and
—modifying the nutrient content of the diet of the animals before making the liver preparation.

These latter approaches are useful as tools to understanding the contributions of certain types of reactive metabolites, and could play an important role in Phase 2 tests, but they are unlikely to be of value in Phase 1 tests.

Although enhanced sensitivity is usually achieved by using cell fractions, there is a danger that the role of metabolites which have little if any relevance to cancer initiation *in vivo* may be emphasized. Such a situation might arise because the metabolite would normally be detoxicated before it could react with essential target macromolecules, or because it would only be formed at cytotoxic dose levels of chemical.

Intact cells may also be used as the active metabolite-generating source. However, it is apparent that isolated hepatocytes tend to show a differential loss of mixed-function oxidases within 24 h of their isolation (Bridges & Fry, 1978*b*). Cell lines derived from hepatocytes are almost entirely deficient in the major xenobiotic metabolizing form of mixed-function oxidase (phenobarbitone P–450) in adult liver, and hence such lines produce an abnormal metabolism of most compounds. A similar problem applies to the use of freshly isolated foetal hepatocytes. The cell lines at present available are quite unsuitable as a feeder layer in short-term tests, except where restricted groups of chemicals are being examined. Freshly derived intact cell preparations from human tissues offer considerable potential as the metabolism system for Phase 2 tests.

*Interfacing the metabolism and response system*

Metabolites formed by the metabolism system must be able to gain ready access to the response system. The physical separation between the two systems should therefore be minimal, and the medium between the two systems should, ideally, allow free diffusion of both polar and non-polar metabolites. This is seldom accomplished in the present tests. Except in the case of aromatic amines, the major reactive metabolites tend to be lipophilic, so that for general application a lipid environment between the metabolism and response systems is more desirable than a polar one. Care must be taken to ensure that changes such as lipid peroxidation or hydrolysis of proteins in the metabolism system as a result of the action of a chemical, do not cause modifications to the response system. This is particularly important in response systems which are critically dependent on the level of nutrient in their environment, *e.g.* bacteria showing dependence for growth on the availability of certain amino acids.

*Selection of response systems*

Many biological changes have been associated with chemical carcinogens: binding of metabolites to DNA, damage to DNA and chromosomes, DNA repair, qualitative and quantitative changes in cell growth, production of abnormal ("marker") proteins, changes in antigenicity of the plasma membrane, differential changes in cellular enzymes and loss of ribosomes from the endoplasmic reticulum. In the light of our reasonably good understanding of how DNA might be involved in cancer initiation by chemicals and the paucity of our knowledge of other mechanisms of cancer initiation and post initiation events, most of the present tests are aimed, perhaps understandably, at determining endpoints associated with DNA and chromosomal changes. In view of our uncertainty about the key events in chemical carcinogenesis it is essential that in the response system particularly, Phase 1 tests should cover a variety of different biological preparations, and that some should allow for initiation events other than direct interaction of the chemical and/or its metabolites with DNA. It is important that in the development of each test the problem of false negatives and false positives is thoroughly researched in order to understand the causes of the limitations. It is essential that each test is validated using a range of chemical structures which *in vivo* have different tissue and species specificities. It is necessary to establish for each chemical a reproducible dose-response relationship and to be able to distinguish between subtle changes likely to be associated *in vivo* with tumour development and gross effects likely to result in cell death. Much more effort must be expended on developing reliable quality control; a single chemical standard is most unlikely to be adequate to control the quality of combined metabolism and response systems.

*The future*

It is to be hoped that the progressive development of improved short-term approaches for detecting and characterizing carcinogens and related compounds will not be inhibited by the natural desire to obtain rapidly a universal short-term test for screening carcinogens.

REFERENCES

Bridges, J. W. & Fry, J. R. (1978*a*) *Carcinogenicity Testing* Ed. Dayan & Brimblecombe. Lancaster: MTP Press. p. 29.

Bridges, J. W. & Fry, J. R. (1978*b*) In *The Induction of Drug Metabolism*. Ed. Estabrook & Lindelaub. Stuttgart: Symposium Medica Hoechst. p. 343.